# The Association of Mental Health Problems With Preventive Behavior and Caregivers' Anxiety About COVID-19 in Children With Neurodevelopmental Disorders

**DOI:** 10.3389/fpsyt.2021.713834

**Published:** 2021-07-16

**Authors:** Kota Suzuki, Michio Hiratani

**Affiliations:** ^1^Faculty of Education, Shitennoji University, Habikino, Japan; ^2^Hiratani Child Development Clinic, Fukui, Japan

**Keywords:** coronavirus disease 2019, preventive behavior, caregivers' anxiety, neurodevelopmental disorder, fears of COVID-19

## Abstract

We examined the association of mental health problems with preventive behavior and caregivers' anxiety in children with neurodevelopmental disorders (NDD) and their caregivers during the coronavirus disease 2019 (COVID-19) pandemic. Data were obtained from 227 pairs of children with NDD and their caregivers in a clinic in Fukui Prefecture, Japan, from October 1 to December 31, 2020. During this period, the activities of children and caregivers were not strongly restricted by the public system. Caregivers' anxiety about children's activities was positively associated with caregivers' and children's fears of COVID-19 and children's depressive symptoms. Children's preventive behavior was negatively associated with children's depressive symptoms. These findings suggested that caregivers' fear of COVID-19 stemmed from worry about the relationship between children's activity and COVID-19 infection, and children might have reflected caregivers' expressions of concern. In schools and clinics, practitioners educate children on how to engage in preventive behavior against COVID-19. Our results support the effectiveness of such practices in mitigating mental health problems in children with NDD.

## Introduction

Lifestyles have been changed by the coronavirus disease 2019 (COVID-19) pandemic; these changes have impacted mental health problems in the general population (1). The mortality risk of COVID-19 is low for children and most caregivers (2), and children are not the main drivers of COVID-19 (3). However, COVID-19 affects the mental health problems of children and their caregivers. For example, the rate of children with a low quality of life increased during the COVID-19 pandemic compared to before the COVID-19 pandemic (4). It has been reported that about 60% of caregivers expressed worry that their child would catch COVID-19 at school (5).

Children with neurodevelopmental disorders (NDD), such as attention-deficit/hyperactivity disorder (ADHD) and autism spectrum disorder (ASD), are at risk for mental health problems (6, 7). Similar risks have also been found in their caregivers (8, 9). In a survey during the COVID-19 pandemic, a higher prevalence of emotional symptoms and conduct problems and fewer prosocial behaviors were found in children with NDD than in those with neurotypical development [NTD; (10)]. Mental health and behavioral problems increased during the COVID-19 pandemic compared to before the COVID-19 pandemic in children with NDD (11, 12). In addition, externalizing behavior was higher in children with NDD than in those with NTD, and that behavior was associated with parenting stress during the COVID-19 pandemic (13, 14). These results suggest that mental health has been worsened by the COVID-19 pandemic in children with NDD as well as in their caregivers.

Educating the public with appropriate information about COVID-19 and related preventive behaviors has been considered important for mitigating children's mental health problems (15). In a previous study, adults who engaged in preventive behavior tended to have better mental health (16). In schools, teachers have provided students with knowledge about COVID-19 and related preventive behaviors (17). In addition, brochures have been developed to educate children with ASD on knowledge and preventive behavior (18). However, there is no evidence that knowledge and preventive behavior are associated with mental health problems in children with NDD.

The behavior and mental health problems of children interact with those of their caregivers (19, 20). In a study from before the COVID-19 pandemic, mothers' anxiety and fearfulness were found to be associated with the anxiety and fearfulness of children (21). Children of caregivers with anxiety disorders tend to be more anxious and fearful than children of caregivers without anxiety disorders (20, 22). Most caregivers worried that their child would catch COVID-19 at school (5). Thus, it is possible that caregivers' anxiety about children's activities related to COVID-19 infection was associated with children's mental health problems.

In this study, we examined the association of mental health problems with preventive behavior and caregivers' anxiety about children's activities in children with NDD and their caregivers. We used data from patients and their caregivers in a clinic in Fukui Prefecture. Japan was in a state of emergency from April 7 to May 25, 2020. During that time, citizens were required to stay home, and children stopped participating in school activities. The number of cases was smaller in Japan than in other developed countries (23). Children continued to participate in school activities starting at the end of the first state of emergency (May 25, 2021). Thus, the activities of children and caregivers were not strongly restricted by public systems. Moreover, Fukui Prefecture is a provincial area of Japan, with a population of approximately 760,000. During the survey period (i.e., October 1 to December 31, 2020), 111 people were infected in the Fukui Prefecture (24). That is, the number of cases was much smaller in Fukui Prefecture than in metropolitan areas of Japan and other countries. On the other hand, the government recommended preventive behavior (25), and the media conveyed information about COVID-19 daily. Thus, we hypothesized that mental health problems of children with NDD and their caregivers would be associated with knowledge about COVID-19, preventive behavior for COVID-19 infection, and caregivers' anxiety about children's activities related to COVID-19, rather than to the COVID-19 pandemic itself.

Mental health problems were evaluated from two aspects: fears of COVID-19 and general mental health problems. The Fear of COVID-19 Scale (FCV-19S) have been developed (26), and was widely used to assess fears of COVID-19. We assumed that the fears of COVID-19 were associated with knowledge about COVID-19, preventive behavior for COVID-19 infection, and caregivers' anxiety about children's activities related to COVID-19. Additionally, the COVID-19 pandemic not only influenced fears regarding the virus itself but also general mental health problems (1). We examined the association of general mental health problems with the variables. In the secondary analysis, we confirmed the relationship between fears of COVID-19 and general mental health problems (26). Further, we thought it useful for clinical practice to clarify types of preventive behavior that children with NDD understood and engaged in. Thus, we examined the difference in items of knowledge about COVID-19 and preventive behavior for COVID-19 infection.

## Methods

### Participants

The participants were children with NDD and their caregivers. We recruited 252 children and 294 caregivers from a developmental clinic in Fukui Prefecture from October 1 to December 31, 2020. All caregivers of the 252 children participated in this study. One pair withdrew their participation after submitting their responses. After excluding incomplete responses, data from 232 pairs remained. Five pairs were excluded because children's IQs were lower than 50. Thus, we used data from 227 pairs in the analyses. The ages of the children ranged from 6 to 18 years. The characteristics and diagnoses are shown in Tables 1, 2. Some children were not diagnosed with clear NDD (e.g., children with low IQ but not intellectual disability and children with ASD tendencies). The caregivers provided written informed consent before participation, and informed consent was obtained from all the children. The study design was approved by the ethics committee of Shitennoji University (2020-17).

**Table 1:** Characteristics of participants.

Child (year)	Mean age	9.96
	Standard deviation	2.46
Caregiver (year)	Mean age (year)	41
	Standard deviation	5.31
Child gender (n)	Boy	189
	Girl	38
Type of caregivers (n)	Mother	196
	Father	31

**Table 2:** Diagnosis of children (n).

	**Comorbidity**		
	**No**	**SLD**	**ID**
ADHD+ASD	37	54	2
ADHD	24	52	3
ASD	12	6	10
SLD	12	NA	NA
ID	5	NA	NA
Other and no clear diagnosis	15	NA	NA

*ADHD, attention-deficit/hyperactivity disorder; ASD, autism spectrum disorder; SLD, specific learning disorder; ID, intellectual disability*.

### Fear of COVID-19

The Japanese version of FCV-19S [FCV-19S-J; (27)] was used to assess the fear of COVID-19 in caregivers. The FCV-19S-J (27) and the original version (26) consists of seven items, and caregivers rated their state using a five-point Likert-type scale (strongly disagree = 1 to strongly agree = 5). Total scores were used for the analysis. A higher score represents a higher degree of fear related to COVID-19.

The expressions of items of the FCV-19S-J were revised to be suitable for children. One item, “I am afraid of losing my life because of COVID-19,” was considered to be too invasive for children with NDD. Thus, we revised this item to “I am afraid of being admitted to a hospital because of COVID-19.” Children rated their state on a five-point Likert-type scale (strongly disagree = 1 to strongly agree = 5). We performed a factor analysis using the maximum-likelihood estimation method. The factor loadings of items ranged from 0.43 to 0.75. Thus, similar to FCV-19S-J, we used the sum of the items to assess fear of COVID-19 in children.

### General Mental Health Problems of Children and Caregivers

The Japanese version of the Birleson Depression Self-Rating Scale for Children [DSRS-C; (28)] was used to assess general mental health problems in children. The DSRS-C consists of 18 items that assess depressive symptoms (29). Children rated their states using a three-point scale (none of the time = 0 to all of the time = 2). We used the sum score of the DSRS-C for the analysis. A higher score indicates a higher degree of depressive symptoms.

To assess general mental health problems of caregivers, we used the Japanese version of the Kessler 6-items Psychological Distress Scale [K6; (30, 31)]. K6 consists of six items that assess depressive and anxiety symptoms. Caregivers rated their state using a five-point Likert-type scale [none of the time = 0 to all of the time = 4; (30)]. The sum score was used in the analysis. A higher score indicates a higher degree of psychological distress.

### Children's Knowledge and Preventive Behavior

Children's knowledge about COVID-19 was tested by seven items: “stay home,” “don't go to crowded places,” “don't speak closely with friends,” “open windows,” “put on a mask outside of the house,” “frequently wash hands,” and “don't touch your eyes, nose, and mouth.” The items were made based on information from the Ministry of Health, Labor and Welfare in Japan in August 2020 (25). Children rated whether the item was related to the prevention of COVID-19 using a three-point scale (incorrect = 1 to correct = 3). We used the mean score of items for the analysis and considered that a higher score represents more appropriate knowledge.

Children's preventive behavior for COVID-19 infection was assessed by themselves and caregivers using seven items identical to those used to assess children's knowledge. Children and caregivers rated how often children engaged in preventive behavior of each item, using a three-point scale (none of time = 1 to always = 3). We used both the mean scores of items rated by children and caregivers for the analysis and considered higher scores to represent more appropriate preventive behavior.

### Caregivers' Preventive Behavior and Anxiety

Caregivers' preventive behavior for COVID-19 infection was assessed using seven items identical to those used to assess children. Caregivers rated adherence to preventive behavior of each item on a six-point scale (behave very incompletely = 1 to behave very completely = 6). We used the mean score of items for the analysis and considered higher scores to represent more appropriate behavior.

Caregivers' anxiety about children's activities related to COVID-19 infection was assessed using six items on children's activities: “school,” “playing with their friends,” “time with their family,” “vacation,” “hospital,” and “all activities.” Caregivers rated their children's activity using a five-point scale (“I am not afraid of the activity at all” = 0 to “I am very afraid of the activity” = 5). We used the mean score of the items for the analysis. A higher score represents a higher degree of anxiety about children's activities related to COVID-19 infection.

### Problematic Behavior

The Japanese version of the Aberrant Behavior Checklist [ABC-J; (32, 33)] was used for assessing problematic behavior of children. The ABC-J consists of 58 items that are classified into five subscales: “irritability,” “lethargy,” “stereotypic behavior,” “hyperactivity,” and “inappropriate speech.” Caregivers rated children's behavior using a four-point scale (no problem = 0 to remarkable problem = 4). We used the sum score for each subscale.

### Analyses

Statistical analyses were performed using the free statistical environment R 3.52 (34). The association between fear of COVID-19 and general mental health problems with other variables was tested using stepwise multiple regression analyses based on Akaike information criteria. For this, we used children's knowledge about COVID-19; their preventive behaviors regarding COVID-19 infection, as rated by both the children and caregivers; caregivers' preventive behavior regarding COVID-19 infection; and their anxiety about children's activities related to COVID-19 infection as independent variables. Additionally, previous studies have shown the association between behavioral characteristics and mental health problems among children with NDD and their caregivers (35, 36). Hence, we added irritability, lethargy, stereotypical behavior, hyperactivity, and inappropriate speech as independent variables. We also added children's age, gender, and IQ and the caregivers' age and type of caregivers (father = 0, mother = 1) as control variables. Finally, the above variables were incorporated in the first model of step-wise regression analyses.

The relationship between children's and caregivers' fears of COVID-19 and general mental health problems was tested using Pearson's correlational test. We evaluated the differences among items in children's knowledge and children's preventive behaviors rated by children and caregivers using one-way analyses of variance (ANOVAs). A Bonferroni test was used for multiple comparisons (α = 0.05).

For correlational analysis and regression analysis, we excluded the data of a participant from the analysis when there was more than one missing value in each variable. When there was one missing value, we calculated the mean values for the data, excluding the missing values, and then used the mean value as the score for the item with a missing value. For ANOVAs on variables, we excluded the data of participants with missing values.

## Results

### Step-Wise Regression Analyses on Fear of COVID-19

Table 3 shows the results of the step-wise regression analysis on children's fear of COVID-19. The final model included caregivers' anxiety regarding children's activities, children's lethargy, children's stereotypic behavior, children's hyperactivity, and children's IQ. We found that the model was significantly associated with children's fear of COVID-19 (*p* < 0.001); children's fear of COVID-19 had significant positive associations with caregivers' anxiety about children's activities and children's stereotypical behavior, whereas it had a significant negative association with children's IQ (Table 3).

**Table 3:** The result of step-wise regression analysis on children's fear of COVID-19.

	***β***	***p***
Caregivers' anxiety for children's activities	1.38	0.001
Children's lethargy	−0.65	0.123
Children's stereotypic behavior	0.99	0.026
Children's hyperactivity	−0.70	0.126
Children's IQ	−0.78	0.045

*R^2^= 0.10, adjusted R^2^= 0.07, F_(5,221)_ = 4.65, p < 0.001*.

Table 4 shows the result of the step-wise regression analysis on caregivers' fear of COVID-19. The final model included caregivers' prevention behavior, caregivers' anxiety for children's activities, children's lethargy, and children's inappropriate speech. The model was significantly associated with caregivers' fear of COVID-19 (*p* < 0.001); there were significant positive associations between caregivers' fear of COVID-19 and caregivers' preventive behavior, caregivers' anxiety about children's activities, and children's inappropriate speech (Table 4).

**Table 4:** The result of the step-wise regression analysis on caregivers' fear of COVID-19.

	***β***	***p***
Caregivers' prevention behavior	0.74	0.005
Caregivers' anxiety for children's activities	1.01	<0.001
Children's lethargy	0.84	0.003
Children's inappropriate speech	0.41	0.137

*R^2^= 0.19, adjusted R^2^= 0.17, F_(4,222)_ = 12.88, p < 0.001*.

### Step-Wise Regression Analyses on General Mental Health Problems

Table 5 shows the results of the step-wise regression analysis on children's depressive symptoms. The final model included children's knowledge, children's prevention behavior, as rated by both self the children themselves and the caregivers, caregivers' anxiety for children's activities, children's irritability, children's lethargy, children's inappropriate speech, and children's age, and the model was significantly associated with children's depressive symptoms (*p* < 0.001). In the model, children's depressive symptoms had a significant positive association with caregivers' anxiety about children's activities, and significant negative associations with children's preventive behaviors, as rated by both the children and caregivers, and children's inappropriate speech (Table 5).

**Table 5:** The result of the step-wise regression analysis on children's depressive symptoms.

	***β***	***p***
Children's knowledge	−0.59	0.109
Children's prevention behavior (self-rating)	−0.95	0.014
Children's prevention behavior (caregivers-rating)	−0.87	0.026
Caregivers' anxiety for children's activities	1.22	0.002
Children's irritability	0.68	0.105
Children's lethargy	0.79	0.055
Children's inappropriate speech	−1.56	<0.001
Children's age	0.72	0.063

*R^2^= 0.18, adjusted R^2^= 0.15, F_(8,218)_ = 5.99, p < 0.001*.

Table 6 shows the results of the step-wise regression analysis on caregivers' psychological distress. The final model included caregivers' prevention behavior, caregivers' anxiety for children's activities, children's lethargy, children's stereotypic behavior, children's inappropriate speech, children's IQ, and type of caregivers. The model was significantly associated with caregivers' psychological distress (*p* < 0.001). In the model, there were significant positive associations between caregivers' psychological distress and caregivers' preventive behavior, children's lethargy, children's inappropriate speech, children's IQ, and mother respondents (Table 6).

**Table 6:** The result of the step-wise regression analysis on caregivers' psychological distress.

	***β***	***P***
Caregivers' prevention behavior	0.59	0.024
Caregivers' anxiety for children's activities	0.37	0.146
Children's lethargy	0.80	0.003
Children's stereotypic behavior	−0.48	0.090
Children's inappropriate speech	0.70	0.022
Children's IQ	0.60	0.020
Type of caregivers (Father = 0, Mother = 1)	0.71	0.005

*R^2^= 0.17, adjusted R^2^= 0.14, F_(7,219)_ = 6.36, p < 0.001*.

### Correlational Analyses

Table 7 shows Pearson's correlational coefficients among fears of COVID-19 and general mental health problems (i.e., depressive symptoms and psychological distress) in children and caregivers. There were significant positive correlations between fears of COVID-19 and general mental health problems in both children and caregivers. However, there were no significant correlations of fears of COVID-19 and general mental health between children and caregivers.

**Table 7:** Pearson's correlation among fears of COVID-19 and general mental health problems in children with neurodevelopmental disorder and their caregivers.

	**1**	**2**	**3**
1) Children's fear of COVID-19			
2) Caregivers' fear of COVID-19	0.16		
3) Children's depressive symptoms	0.20^*^	0.03	
4) Caregivers' psychological distress	0.10	0.30^***^	0.07

* *p < 0.05*,

^**^p < 0.01,

*** *p < 0.001 (Bonferroni correction)*.

### Children's Knowledge About COVID-19 and Preventive Behavior for COVID-19 Infection

Table 8 shows means and standard deviations of children's knowledge about COVID-19 and preventive behavior for COVID-19 infection. One-way ANOVAs showed significant main effects of items (children's knowledge about COVID-19: *F*_(6, 1356)_ = 31.44, *p* < 0.001; children's preventive behavior rated by the children: *F*_(6, 1344)_ = 60.72, *p* < 0.001; children's preventive behavior rated by caregivers: *F*_(6, 1326)_ = 101.46, *p* < 0.001). In terms of children's knowledge about COVID-19, Bonferroni tests showed that scores were significantly higher on “don't go to crowded places,” “put on a mask outside of the house,” and “wash hands frequently” than on the other items. For children's preventive behaviors rated by both the children and caregivers, Bonferroni tests showed that scores were significantly higher on “put on a mask outside of the house,” and “wash hands frequently” than on other items. Bonferroni tests also showed that scores were significantly higher on “put on a mask outside of the house” than “wash hands frequently.” In addition, Bonferroni tests showed that scores were significantly higher on “stay home” than “don't go to crowded places.”

**Table 8:** Means (standard deviations) of children's knowledge about COVID-19 and prevention behavior for COVID-19 infection.

	**Home**	**Place**	**Closeness**	**Window**	**Mask**	**Hand**	**Face**
Knowledge	2.65 (0.62)	2.90 (0.34)	2.49 (0.69)	2.62 (0.65)	2.90 (0.36)	2.94 (0.24)	2.59 (0.60)
Prevention behavior (self-rating)	2.16 (0.71)	1.99 (0.82)	2.03 (0.76)	2.08 (0.78)	2.87 (0.41)	2.62 (0.61)	2.09 (0.74)
Prevention behavior (caregivers-rating)	1.95 (0.74)	1.84 (0.82)	1.82 (0.75)	1.85 (0.69)	2.84 (0.48)	2.47 (0.68)	1.71 (0.64)

*Home: “stay home,” Place: “don't go to crowded places,” Closeness: “don't speak closely with friends,” Window: “open windows,” Mask: “put on a mask outside of the house,” Hand: “frequently wash hands,” Face: “don't touch your eyes, nose, and mouth.”*

## Discussion

We examined the association of mental health problems with preventive behavior and caregivers' anxiety in children with NDD and their caregivers during the COVID-19 pandemic. There were significant positive correlations between fear of COVID-19 and general mental health problems in children with NDD and their caregivers. Thus, we confirmed the relationship between fear of COVID-19 and general mental health problems in children with NDD and their caregivers. In addition, caregivers' anxiety about children's activities was positively associated with fear of COVID-19 in caregivers. Children have a low mortality risk of COVID-19 (2), and are not the main drivers of the pandemic (3). In spite of this evidence, most caregivers worried that their child would catch COVID-19 at school (5). Our results indicated that caregivers' fear stemmed from worry about the relationship between children's activity and COVID-19 infection.

Caregivers' anxiety about children's activities related to COVID-19 infection was positively associated with children's fear of COVID-19 and depressive symptoms. A previous study conducted before the COVID-19 pandemic indicated that caregivers' anxiety is associated with their children's anxiety (20–22). It is possible that children reflected caregivers' expressions of worry about the relationship between children's activity and COVID-19 infection. In addition, we hypothesized that caregivers' anxiety about children's activity might be related to over-involvement in children's preventive behavior. A systematic review showed that caregivers' over-involvement is associated with anxiety and depressive symptoms (37). Therefore, we suggest that mental health problems in children with NDD were affected by caregivers' expression of worry and over-involvement.

Children's preventive behavior was negatively associated with children's depressive symptoms, which indicates that children who acquired preventive behavior for COVID-19 tended to have lower depressive symptoms. Most children with NDD engaged in preventive behavior of “put on a mask outside of the house” and “wash hands frequently.” In schools, teachers have educated students on knowledge and preventive behavior for COVID-19 (17), and educational methods have been developed for children with NDD (15, 18). Our results support the idea that these practices mitigated children's general mental health problems during the COVID-19 pandemic.

In contrast to children's preventive behavior, caregivers' preventive behavior was positively associated with caregivers' fear of COVID-19 and psychological distress. Inconsistent results were reported in a previous study in which preventive behavior was associated with better mental health (16). Japanese people had been required to engage in preventive behavior for more than half a year at the time of the survey. The number of cases was small in Fukui Prefecture during the survey period (24). Most people did not strictly engage in preventive behaviors such as “stay home.” We hypothesize that “excessive” preventive behavior was a burden for caregivers, which might have exacerbated mental health problems.

Children's IQ was negatively associated with children's fear of COVID-19, whereas it was positively associated with caregivers' psychological distress. Children with high IQs can effectively acquire knowledge about COVID-19 and can freely behave independently of caregivers, which might reduce children's fear of COVID-19 and might enhance caregivers' psychological distress. In addition, children's inappropriate speech was negatively associated with children's depressive symptoms, whereas it was positively associated with caregivers' psychological distress. One item, “talks excessively,” is included in inappropriate speech (32), and it is also one of the factors that can increase the spread of COVID-19 infection. Thus, we considered that caregivers of children with a higher score of inappropriate speech worried that the children would spread COVID-19. On the other hand, we speculated that a higher score for inappropriate speech represented a low level of worry about COVID-19 infection; thus, children with higher scores might not feel burdened.

In the statistical analysis, the fears of COVID-19 and general mental health problems were used as dependent variables. However, the associations between the dependent and independent variables were unclear. Therefore, longitudinal data are necessary to test the causality. Moreover, since the COVID-19 pandemic is on-going, it would difficult for the future studies to confirm the present findings. We expect clinicians to report their practices regarding awareness of preventive behavior and interventions for caregivers, which might partially support the present findings. In addition, most variables were based on self-reported scales; thus, the findings are biased. Especially, preventive behavior regarding COVID-19 might have the tendency to be scored higher owing to government recommendations and media information regarding COVID-19. In the all models of the step-wise regression analyses, the coefficients of determination were <0.20, which indicates that the other variable might more strongly explain the fears of COVID-19 and general mental health problems. Furthermore, the results might not be unique to children with NDD and their caregivers because only clinical data were available. We acquired patient- and caregiver-data from a single clinic. Therefore, our results are not representative of children with NDD and their caregivers. Additionally, patients with severe mental health problems could not participated in this study. Thus, the present findings were based on the data from participants who could respond to the questionnaire. Finally, the severity of the COVID-19 pandemic is fluctuating and differs across countries and regions. It is, thus, difficult to obtain representative results; Fukui Prefecture is a provincial area, and the number of cases was small during the survey period (24). However, despite the above limitations, we believe that our results are valuable as it analyzes these issues in the context of the pandemic.

We examined the association of mental health problems with preventive behavior and caregivers' anxiety in children with NDD and their caregivers during the COVID-19 pandemic. Caregivers' anxiety about children's activities was positively associated with caregivers' and children's fears of COVID-19 and children's depressive symptoms. These results suggest that caregivers' fear of COVID-19 stems from worry about the relationship between children's activity and COVID-19 infection and that children might reflect caregivers' expressions of concern. Children's preventive behavior was negatively associated with children's depressive symptoms. In schools and clinics, practitioners educate children to engage in preventive behavior against COVID-19. Our results support the effectiveness of such practices in mitigating general mental health problems in children with NDD.

## Data Availability Statement

The datasets presented in this article are not readily available because the data that support the findings of this study are available on request from the author, and approval by the ethics committee of Shitennoji University. The data are not publicly available because no informed consent was given by the participants for open data sharing. Requests to access the datasets should be directed to Kota Suzuki, kt.suzuki@hotmail.co.jp; ktsuzuki@shitennoji.ac.jp.

## Ethics Statement

The studies involving human participants were reviewed and approved by the ethics committee of Shitennoji University. The caregivers provided written informed consent before participation, and informed consent was obtained from all children.

## Author Contributions

KS contributed to the study design, interpreting the data, and writing the manuscript. MH contributed to the study design, data collection, interpreting the data, and writing the manuscript. Both authors contributed to the article and approved the submitted version.

## Conflict of Interest

The authors declare that the research was conducted in the absence of any commercial or financial relationships that could be construed as a potential conflict of interest.
